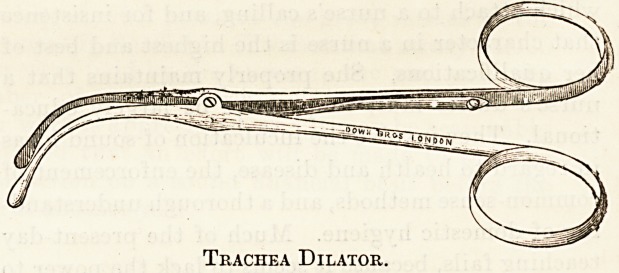# The Hospital. Nursing Section

**Published:** 1905-12-02

**Authors:** 


### The Hospital.

### Nursing Section.

Contributions for "THE HOSPITAL," should be addressed to the EDITOR, 'THE HOSPITAL" NURSING SECTION, 28 & 29 Southampton Street, Strand, London, W.C.

No. 1,001.—VOL. XXXIX. SATURDAY, DECEMBER 2, 1905.

### Notes on News from the Nursing World.

### THE ABSENCE OF A NURSE ON THE ROYAL COMMISSION.

THE promised Royal Commission on the Poor Laws has at last been constituted, and the names of the members were officially announced on Wednesday. We welcome, in the interests of women, the appointment of Mrs. Bernard Bosanquet, Mrs. Sidney Webb, and Miss Octavia Hill. But we regret that no lady of prominence in the nursing world who combines wide experience with practical knowledge, has been placed on the Commission. In an investigation which must necessarily cover the whole range of the system of nursing in the Poor-law Infirmaries, it is a matter for regret that no fully trained nurse has been invited to take part. It is obvious that a body charged with the task of inquiring into the working of the laws relating to the relief of poor persons, a large proportion of whom are patients in the infirmaries or sick wards of the workhouses, would be all the stronger for her presence and is therefore all the weaker for her absence.

### PRIVATE CONFERENCES ON PUBLIC QUESTIONS.

A CONFERENCE of "Queen's" Superintendents was held at the Hotel Windsor on Wednesday, and a report of the proceedings would have appeared in our columns to-day, but in spite of the special interest attaching to the presentation of a cheque and address to Miss Peter, the late general superintendent of the Jubilee Institute, representatives of the Press were excluded. We were courteously informed that an account of this ceremony and of the discussion would be sent to us afterwards, but we think that it would be far more satisfactory if conferences in which not only Queen's nurses throughout the country, but all the subscribers to the Jubilee Institute, are concerned, were not treated as "strictly private." Such questions as "How inspection may be made of the most use," "How best to utilise outside help," and "The relation of Queen's Nurses to the local supervising authorities under the Midwives Act," were, we believe, debated on the occasion, and we can see no adequate reason why the debates should have been held with closed doors. Private conferences on public questions by organisations dependent for existence upon public money are out of date.

### THE MILITARY NURSING SERVICES.

WE are officially informed that Miss J. S. G. Gardner has been appointed staff nurse and Miss C. W. Jones staff nurse provisionally in Quee[illegible] Alexandra's Imperial Military Nursing Servic[illegible] Miss E. M. Denne, sister, has been poste[illegible] to the Royal Herbert Hospital, Woolwich Miss E. L. McAllister, staff nurse, to th[illegible] Royal Victoria Hospital, Netley, and Mi[illegible] A. A. Steer, staff nurse, to Queen Alexandra Military Hospital, Millbank, on their return from Indian troopship duty; Miss B. N. Daker, sister, has been transferred from Queen Alexandra Military Hospital, Millbank, S.W., to s.s. *Plassy* for Indian troopship duty. The following ladies have been appointed nursing sisters in Queen Alexandra's Imperial Military Nursing Service for India: Miss G. R. N. Percival, Miss E. A. Todd, Mrs. Stanley Clay, Miss A. O'Brien, and Miss H. E. Hornsby.

### THE ANNUAL DANCE AT GUY'S.

ON Wednesday last week the residents of Gu[illegible] Hospital gave their annual dance, which t[illegible] place in the beautiful court-room. The fog [illegible] the previous day which threatened to throw a glo[illegible] over everything had disappeared and the even[illegible] was fine and the air not too cold to admit of o[illegible] windows. Commencing at 8.30 P.M., dancing c[illegible] tinued until 1 A.M. If outward appearance may [illegible] taken as proof, everybody enjoyed themselv[illegible] The brightness of the whole scene, the animati[illegible] of the dancers, the complacency of the onlookers and the excellence of the music, produced a gene[illegible] air of happiness which spoke sufficiently for t[illegible] assured success of the evening and the thorou[illegible] enjoyment of all present. Many of the sisters of t[illegible] hospital were present and they entered heartily in the spirit of the evening and enjoyed it perhaps m[illegible] than any, because of the thorough relaxation afforded from their duties.

### THE STANDARD OF MENTAL NURSING.

IN presenting the badges of the Medico-Psyc[illegible] logical Society to the nurses and attendants of Glasgow Royal Asylum, and the nurses with ce[illegible] ficates for sick-room cookery, Lady Ure Prim[illegible] made some appropriate remarks on the import[illegible] of elevating mental nursing to the highest pos[illegible] level. She thinks that for nursing mental dis[illegible] the services of gentlewomen, using the word i[illegible] broadest sense as meaning women of sympathy, [illegible] and good heart, are specially wanted. We [illegible] satisfied that this is the right view of the posi[illegible] and we should be glad to see more of the cla[illegible] whom Lady Ure Primrose refers coming for[illegible]

to undertake work in a field which offers scope for the highest qualities of both heart and brain.

### HONOURS FOR AN IRISH MATRON.

An interesting function took place at the Palace, St. Stephen's Green, Dublin, last week, when several members of the Committee of St. Patrick's Nurses' Home assembled in order to take part in the presentation to Miss F. Franceys Howell, late Superintendent of the Home, of an address and testimonial commemorating her fourteen years of service. The Archbishop of Dublin, who is President of the institution, asked Miss Howell to accept the address and testimonial as marks of the esteem in which she was held by all connected with the Home. She had gained the confidence of those with whom she worked and had handed over the Home to her successor in a state of complete efficiency both for nursing the poor in their own homes and for training nurses for the work. Miss Howell, in appropriate language, expressed her warm thanks for the kind words of the Archbishop and her appreciation of the presentation.

### THE CENTRAL MIDWIVES BOARD AND ASTON UNION INFIRMARY.

The refusal of the Central Midwives Board to approve the Aston Poor-law Infirmary as a training school for midwives on the ground that the structural and general conditions of the infirmary are unsatisfactory, has prompted the guardians to arrange for an examination by a recognised expert of the maternity wards, and of the system of nursing and of training nurses. The issue of the report will be awaited with more than local interest. There is a strong feeling, extending beyond past and present nurses at the Aston Poor-law Infirmary, that the decision of the Central Midwives Board, in this instance, is a mistake.

### HOW NOT TO APPOINT A PARISH NURSE.

An interesting letter on the subject of the parish nurse in relation to medical men appears in a West of England paper from "A Parish Surgeon," who mentions that, feeling the want of a nurse in the village, he publicly drew attention to the need. Shortly afterwards the vicar of a neighbouring parish formed a committee, engaged a nurse, and promulgated a code of rules without any reference to him or to any other medical man. The surgeon is now asked to employ the nurse, whom he does not know, and over whom he has no control. Her patrons, moreover, are endeavouring to obtain for her cases of midwifery at 4s. each, without considering that in deliveries attended with danger it is to the surgeon that the nurse would have to turn for assistance. It is naturally annoying to a medical man, who is anxious to work with the nurse for the benefit of the sick poor, to find himself entirely ignored in the matter; and it is also hard for the nurse, who is probably equally anxious to work with the doctor, to find at the outset that he is seriously prejudiced against her. In this instance it is the committee who are entirely to blame. They ought to know that it is essential before engaging a parish nurse to secure the co-operation of the doctor under whom she is expected to work.

### A MATRON ON NURSING.

As usual, the annual prize-giving to the nurses connected with the Bristol Hospital for Sick Children and Women was attended by a large number of supporters and friends of the institution. The first prize, presented by the President for general proficiency at the close of two years' training, was won by Nurse Gotto, and the second by Nurse Richards. Other recipients of certificates of merit and prizes were Nurses Scott, Eleanor Richards, Medway, Beamish, Williams, Lewis, Sheldon, Hill, and Millard. The matron, Miss Mattick, in seconding a vote of thanks to Miss Clifford for distributing the awards, urged nurses to be obedient to those in authority, kind, sympathetic, and patient. It was said that nursing made a woman hard, unsympathetic, and less refined, but she maintained that the contrary was the case, and that nursing, taken up in the right spirit, made a woman more kind and sympathetic than she would otherwise have been. In conclusion, Miss Mattick acknowledged the assistance which she had received from the sisters.

### QUEEN'S NURSES AT BRIGHTON.

The proceedings at the ninth annual meeting, last week, of the Brighton, Hove, and Preston District Nursing Association, were notable for an admirable address by Dr. Newsholme, who at considerable length spoke of the practical value of the work done by the Queen's nurses. He affirmed that it was valuable not only because they helped people to help themselves, keeping many chronic cases out of the hospitals and workhouse infirmaries, but also because of its educational side, for the nurses assisted in dispelling the illusions of the poor in connection with sick nursing, and inculcated the great principle of cleanliness, ventilation, and the early recognition of disease. We are glad to learn from the report that the finances of the Association are on a better footing, the increase in the income last year being £260. The deficit is now small, and as Mr. E. A. Villiers, M.P., said at the meeting, it does not imply that the Association is on the brink of dissolution. It is, in fact, such a useful organisation that we confidently anticipate that by the end of another year the balance will be on the right side.

### NO RELIGIOUS QUESTION FOR POOR-LAW NURSES.

We are glad to learn that, after a full discussion, the Barrow-in-Furness Guardians have assented to a motion on the part of Father Miller, "That this Board disapprove of any person being debarred from the office of nurse on account of her religion." It came out clearly enough that certain Guardians wished to ascertain the religion professed by the nurses, and it was as clearly demonstrated that they objected to the employment of Roman Catholics. The result, however, that no applicant for an appointment is to be interrogated by the Board as to her religion, is not only satisfactory, it is also in accordance with the regulations of the Local Government Board, to which we alluded last week.

### A WELL-MANAGED RURAL NURSING ASSOCIATION.

It is a feather in the cap of the Heathfield and Waldron Nursing Association that, in addition to paying its own way, it was able last year to subscribe £10 to the Sussex County Association. This is not, as may perhaps be imagined that because Heathfield is a delightful and prosperous looking village, there are few sick poor to visit. On the contrary, the two nurses paid upwards of 2,756 visits in the twelve months to nearly 140 patients. In order to pay these visits, however, they have often to traverse many miles, and the conditions of work are by no means easy to fulfil. It is credible to the Committee of the Association and to the people residing in the district that the fund is made up of the modest subscriptions of the many, rather than as is too often the case, of the large contributions of the few.

### AN APPOINTMENT IN NATAL.

It is interesting, in the light of a question which is asked us to-day about the value of the training at Crumpsall Infirmary, Manchester, to learn that Miss Esther Watts, who was trained at Crumpsall, and was afterwards engaged in private nursing in and near London for several years, has been appointed nursing sister at Hilton College, near Maritzburg, one of the leading public schools of Natal. As a matter of fact, many nurses trained at Crumpsall, which has for years been in the van of poor-law nursing, have subsequently filled important posts.

### NURSES AND THE MISSIONARY UNION.

On Friday evening last the nursing staff of St. Mary's Hospital, Paddington, were invited to attend a meeting in the Board-room in connection with the work of medical missions abroad. The chair was taken by Dr. Handfield Jones. Miss Poulter, of China, gave an interesting account of some of her experiences. She mentioned that she had often had to act the part of anæsthetist to her sister, who is a doctor, while the latter performed operations practically single-handed, and stated that nurses are much needed for medical mission work in China. There was an interval between the speeches, when a party of nurses handed refreshments round. Mr. T. Jays, M.R.C.S., then gave a graphic description of his work in West Africa. A branch of the Missionary Union has been started in the hospital, under the supervision of the chaplain, with Sister Bale as honorary secretary.

### CROYDON INSTITUTE.

It is now a quarter of a century since that excellent institution, the Croydon Nurses' Institute, was formed, and the twenty-fifth annual report, just issued, shows that it continues to be enormously appreciated in the great and growing town under the shadow of the metropolis. There are now six district nurses always at work, two of whom are maintained by private people for special parishes, one for the Parish Church district and another for Shirley and part of Addington. The other four nurses paid during the past year 8,563 visits, and in view of the needs of the poor in the town we agree with the Committee that the time has come for an augmentation of the staff. The District Nursing League, which was formed last year, in order to obtain fresh members, has already been very helpful, and no doubt its members will consider it a point of honour to secure the additional income requisite to justify the engagement of another nurse.

### A DEFICIT AT SHEFFIELD.

The second annual report of the Sheffield Queen Victoria's District Nursing Association was adopted at a meeting in the Town Hall, on the motion of Lady Alexander Wilson. We regret to learn that the year, which began with a deficit of £90, ended with one of £204. The organisation has received liberal support from the local boards of guardians and other bodies, but a special appeal to the clergy and ministers of the various chapels has not so far met with an entirely satisfactory response. Perhaps the speech delivered by Sir Dyce Duckworth, as a representative of the Royal College of Physicians on the Council of Queen Victoria's Jubilee Institute, may have a stimulating effect. He confessed that the Council were disappointed that the great needs of the sick poor had not obtained a more general recognition, but acknowledged with pleasure the great success of the movement in Sheffield. The fact that nearly 34,000 visits were paid by the nurses in the past year, the number of patients being 964, shows how much their work is appreciated, and it may be reasonably concluded that an effort will be made to put the organisation on a sound financial basis before the close of another year.

### NOT A TRAINED NURSE.

The woman who last month cut the throat of a baby in a private nursing home in Brighton and was described as "a nurse who had been in training in London," was tried at the Sussex Assizes last week and found guilty of murder but not responsible for her actions. The description was, however, inaccurate; the unfortunate person, who seems to have been the victim of religious mania, was a children's nurse who prior to her mental breakdown had been a servant in one family for ten years.

### OUR CHRISTMAS DISTRIBUTION.

The following is an extract from one of the numerous letters we have received from matrons anxious to participate in our Christmas distribution of clothing: "I am writing to ask if you would be so very kind as to give us a parcel of clothing when distributing at Christmas. We get a great many very, very poor patients, who would be benefited by a warm garment, and most grateful." We have to acknowledge the receipt of parcels from Miss E. J. Henslowe, Carrington House, Weymouth, and M. E. G., Penrith. All contributions should be addressed to the Editor, 28 and 29 Southampton Street, Strand, London, W.C., and should have "Clothing Distribution" written outside.

### The Nursing Outlook.

"From magnanimity, all fear above;
From nobler recompense, above applause,
Which owes to man's short outlook all its charm."

### "A STUDY IN NURSING."

Miss Pringle, who from long service and great experience must be regarded as one of the pioneers of modern hospital nursing, has written a book with the above title (Macmillan and Co.) which the head of every nurse-training school, and indeed every nurse, should procure and ponder. Miss Pringle was lady superintendent of the Royal Infirmary, Edinburgh, in the 'seventies, and she has occupied the important positions of superintendent of the Nightingale Training School for Nurses and matron of St. Thomas's Hospital. Miss Pringle, indeed, is one of the quiet, thoughtful, capable leaders of the nursing world, who has done yeoman service without looking for thanks or expectation of reward.

Miss Pringle stands for self-respect, hard work, the constant recognition of the responsibilities which attach to a nurse's calling, and for insistence that character in a nurse is the highest and best of her qualifications. She properly maintains that a nurse's duties must in practice be largely educational. They involve the inculcation of sound ideas in regard to health and disease, the enforcement of common-sense methods, and a thorough understanding of domestic hygiene. Much of the present-day teaching fails, because it seems to lack the power to impress the taught with the sense that knowledge of this kind, to be useful, must be reduced to practice. What, for instance, is the use of knowing the proportions of carbonic acid and oxygen in pure air, unless the nurse secures that the air her patient breathes is continuously renewed, and that the sick chamber is sweetened by systematically ensuring the proper airing of all bedding, and of thorough cleanliness in all the surroundings of the sick? Miss Pringle also points out that a nurse who indulges in tight lacing and high heels can have benefited little by the lectures she may have heard on physiology and anatomy. For practical purposes, a fully trained nurse who has passed her examination with success must fail in her work, if she is not able to enforce by example, and to inculcate in her patients by practice, that enough warm underclothing should be worn, that all food should be digestible and taken at proper intervals, and that some manual work every day, with regular outdoor exercises, and enough but not too much bed, are essentials to health for both nurses and patients.

Miss Pringle has a quaint and dry humour. The little digs which she gives, perhaps unconsciously, at many ways known to matrons and sisters are really funny, and will provide entertainment for many a hospital worker of experience. The chapter devoted to the matron's duties is one of the best in the book. How true it is that the matron's part in a great hospital, as to the larger share of the many duties which she has to organise, is not to do them herself, but to apportion them among her staff of assistants, and by her oversight to know that they are done. We welcome too Miss Pringle's warning against people in a large establishment ready to "carry tales." It is the hand of experience which tests their value by suggesting that the accused should be sent for, and dismisses the "carry tale" when in such circumstances she stipulates that her name is not to be mentioned, as she "has told matron in strict confidence." It is quite true that the bearers of reports "may have goodwill but not very good sense." The matron, however, should depend upon her own eyes and ears alone. Any institution where the "carry tale" is tolerated must be an unhappy place for the staff, and an inefficient establishment to boot. No really good matron remains in office unless she is able every day, morning, afternoon, evening, or night, to visit some of the wards, all in turn though not in regular order. These "will not be visits of vigorous inspection, but of friendly insight and quiet observation." Every able superintendent and matron will agree that holders of these important offices should keep a strict hand over themselves in the matter of partiality, "an evil it is not easy to steer clear of"!

Miss Pringle's appreciation of the high functions and delights of the ward sisters, who are also good and true women, is in many ways excellent. But the whole of this book should be read, as we have said, by every hospital worker. We are glad to welcome the caution given against "a too intense seriousness in hospital work." The labours of hospital workers grow easier through use, and their methods improve by experience. By experience the mind grows more at liberty for sympathy, joy and sorrow, for as some are dying others are recovering or having their pains relieved, and in regard to all the inmates of a hospital or a sick-room there is the ennobling sense of helpfulness. "The winning ways of the children, the brightness of the young life in the nursing school, the quaint things that are said, and the amusing things that happen, somewhere, in the hospital every day, break in on the seriousness and counteract it." Indeed, Miss Pringle holds, rightly, that each hospital would do well to have a big MS. book chained to a desk, in which should be chronicled, as they occur, things grave and gay, that seem too good to be lost.

### Laryngeal Diphtheria.

By Mrs. HICKS, Formerly Matron of the Evelina Hospital for Sick Children.

### IV. INTUBATION AND TRACHEOTOMY.

(*Concluded from page* 101.)

The patient is dressed in a flannel night-gown, cut square in front, and fastening at the back; a piece of thin pliable mackintosh is pinned round the *décolleté* part of the gown. The elbows must be fixed, either by special arm-stays or splints, so that the patient cannot possibly touch the tube. Straps to secure a permanent horizontal position are applied, and the steam-kettle turned on. The bed-side locker is furnished with a dish containing the dilator and the pilot belonging to the patient's tube; a good supply of small pieces of muslin or gauze with which to quickly catch any discharge that is being coughed up.

It is a good plan to lay a single layer of gauze over the orifice of the tube, as this helps to retain expectoration which otherwise might easily be sucked back. The nurse must be untiring in removing this expectoration, and always be careful to examine it for pieces of membrane. A porringer with some lotio carbol. 1-20 should be at hand to receive the soiled swabs. If there is a great deal of discharge, and the tube does not seem clear, it will be the nurse's duty to remove the inner tube and clean it. This is done by steadying the outer tube with the left hand, and withdrawing the inner tube with the right hand. With Durham's tube you simply pull the inner tube out gently, whereas with Parker's you must remember to first turn the key at the upper part. The tube is cleaned with pieces of clean rag dipped in hot saturated solution of sod. bicarbon. (of which there should always be a plentiful supply), and pushed through the lumen of the tube with a probe, the process being repeated with fresh rags until the tube is perfectly clear again.

Then, standing on the patient's right side and facing him, the nurse reinserts the tube without any difficulty, and remembers to turn the tap if dealing with Parker's tube.

Feathers to clean out the tube *in situ* are dangerous, and should only be used by the doctor, or by his orders, but a feather is a convenient medium for dropping a minim or two of warm saturated solution of sod. bicarbon. into the trachea, a very effective proceeding in cases when the patient makes vain efforts to get rid of the expectoration, which is too dry to be coughed up.

If this moistening and repeated clearing out of the inner tube is unsuccessful, and the child's colour bad, and the intercostal muscles are sucked in during inspiration, we must send for the doctor, who will remove both tubes, we being ready to hand the dilator and to clean out and retape the tubes quickly.

At times a child will suddenly turn blue, get extremely restless, and seem to be suffocating. In this case the inner tube may be entirely blocked by a piece of membrane and must be removed at once. If taking out the inner tube gives no relief, the nurse must cut the tape, pull out the remaining tube, and this may be followed by the expulsion of a piece of membrane too large to have passed through the lumen of the tubes. I have seen a child get into this extreme condition, and the withdrawal of the tubes set free a portion of membrane, showing the complete cast of the trachea and its bifurcation. In an emergency like this there is no time to wait for help; only the nurse's quick acting and sense will save the patient's life. Of course she must ring the bell for her superior as soon as she can spare a hand for the purpose, but after the membrane is expelled there will often be no immediate cause for hurry, or even interference, the relief to the patient's respiration being so great that the dilator may not be wanted, and the tubes omitted permanently.

If the patient progresses favourably, a first attempt to leave out the tube is generally made on the second or third day, a light gauze dressing fixed with a turn of bandage placed over the incised trachea. The tube will often have to be re-inserted at once, and when it has to be worn for a long time, the less irritating india-rubber tube may be substituted for the metal one. This only being a single tube, will have to be changed every twelve hours at least, and often more frequently, for cleaning purposes, so a second similar tube should always be at hand to replace the other, which should be cleared out, boiled, and retaped ready for use.

It is sometimes a great trouble to accustom the patient to do without the tube, even if the larynx has quite recovered, and in this case a tube which has an additional opening at its bend towards the larynx is occasionally successful. We can then block up the outer orifice by degrees, and if we find that the child uses the larynx all right, the doctor will often remove the tubes under an anæsthetic, and so get the child over the moral difficulty.

As in the case of omitting the tube in intubation patients, the nurse may by her influence do a lot to tide the patient over this trying period of discomfort and nervousness.

Trachea Dilator.

The dilator has to be used occasionally by a nurse; and it is done by steadying the patient's head with the left hand, introducing the dilator into the aperture forward and downward, with the blades approximated. When the blades have entered the trachea lengthways we open them by pressing the two parts of the handle together. Often when a tube that has been left out for some time has to be reintroduced the orifice of the wound will be found to have shrunk meanwhile; the dilator has to be inserted for gently re-widening the aperture, and so allow the tube to slip in again.

The tracheotomy wound is sponged over with warm boric lotion, and the gauze changed as often as possible, so as to minimise the danger of infection. Boric powder, iodoform, and various ointments may be used; but we must avoid the latter with india-rubber tubes, as this fabric is spoilt by contact with grease.

The mouth of the patient requires the same constant care as that of intubation patients.

Feeding tracheotomy patients presents few difficulties. As a rule they are able to swallow without trouble, and we must try to make them take as much nourishing food as possible, so as to enable them to fight the disease. We also give as much water or other simple fluids as we can get the patient to take in order to dilute the bacterial poiso[illegible] Only in a very few instances will the nasal tube have to [illegible] used.

Tracheotomy patients are even more susceptible to cold than their intubation brothers, the external air reaching the trachea direct, without having been previously warmed in the mouth, nose, and upper air passages. Hence special pains must be taken that the steam-kettle be always in full working, so as to supply a continuous substitute for the natural warming process.

Unfortunately, even with every care, lung complications, bronchitis, and pneumonia are frequent; and septic broncho-pneumonia, produced by aspirating foul discharges, is a common cause of a fatal termination.

### The Nurses' Clinic.

### THE DISTRICT NURSE AND PARALYSIS. BY MISS M. LOANE.

Owing to the length of time that people may, and do, live after becoming helplessly paralysed, cases of hemiplegia and paraplegia seem to be common among district patients, and for some unknown reasons they and their relatives are often among the worthiest persons with whom the nurse comes into contact, and anything that she can do to help them appears in the light of a privilege as well as a duty.

In severe cases of paralysis every effort should be made to obtain the loan of a water-bed for the patient, and careful instructions must be given as to the way in which it should be filled, and the precautions necessary to prevent any injury to such a costly nursing requisite. The water-bed rarely arrives until the patient is seriously ill, and while the needful arrangements (which can scarcely take less than 1½ hour) are being made, the patient should be lifted on to another bed which has been drawn up side by side with his own. As the weight of a full-sied water-bed may easily amount to about 200 lbs., it is desirable to strengthen the wire mattress by placing five fracture boards across the framework. The bed should then be laid upon the ordinary mattress and filled with water mixed to a temperature of about 90° F., the whole of the water having been previously boiled in order to sterilise it. Water should be added until the bed is so full that a heavy person pressing both hands down on it is unable to make the upper and under sides of the bed touch one another. A sheet should then be placed over it and covered with a mackintosh draw-sheet. The under sheet, draw-sheet, and mackintosh must be firmly fastened to the edge of the mattress with coarse safety pins, care being taken not to puncture or even scratch the water-bed.

The water-bed must be entirely emptied at least once in three months, and in very cold weather a few gallons should occasionally be drawn off and replaced by warm water. If the patient is well enough to get up for a short time, the water-bed should be covered with a blanket and kept warm by two bottles or tins filled with warm (not boiling) water. If this is not done, the patient, returning to a cold bed, might easily take a chill.

The great advantage of a water-bed is that the danger of bed-sores is reduced to a minimum, and heel caps, elbow caps, etc., can be dispensed with; the one drawback is the greater difficulty in lifting the patient, but in any case two persons would always be necessary for this part of the work.

The bed, as in all cases where the patient is heavy or helpless, should be narrow, and a pulley must be fixed to the foot-rail so that the patient can have the mental and physical relief of occasionally shifting his position. If the patient be completely bedridden the bedstead should be high, to reduce the fatigue of nursing and to give the invalid more air and light, and, if possible, the bed should be so placed that there is a window on the left-hand side. If the patient requires attention at night a small extra bed can be brought into the room, but it should be removed in the daytime in order to leave more air and space.

Paralysed patients frequently suffer from incontinence of urine. A vessel of suitable shape should be wrapped round with absorbent wool or tow and placed on a small wedge-shaped pillow left constantly in position. As there is consequent difficulty in keeping the patient dry, the back must not only be well washed and rubbed with methylated spirit, but greased with zinc ointment. There is constant danger of bed-sores, and the relatives must be advised to buy supplies of methylated spirit, powder, and ointment, as these articles are needed every day and can be bought cheaper in large quantities.

A paralysed patient needs washing all over every day with soap and warm water, and special attention must be given to armpits, groins, and all folds of the skin. As this part of the work takes at least an hour, it is improbable that the district nurse will be able to do it herself more than twice a week, and she should therefore take the first opportunity of teaching the relatives how to do it properly. One of the chief points to be insisted on is that the patient shall be wiped with dry, well-warmed towels.

If the hands are at all contracted a ball of absorbent wool must be placed in the palm, and a little pad of wool between the fingers. The finger nails should be kept very short, as if allowed to pierce the palm painful wounds may result. It is desirable that bedridden patients should wear their hair short, but this is, of course, a matter for the patient to decide.

The patient should have what is popularly called "a workhouse shirt." It is a nightshirt made of flannel or flannelette, open at the back, and only fastened by a few strings. It is easily kept dry and put on and off without much difficulty.

If practicable, the patient should be got up every day, wrapped in blankets—where no dressing-gown can be obtained—placed in an easy chair by the fire, and encouraged to read, smoke, or work. The change of position not only breaks the monotony of the day and reduces the risk of bed-sores, but it enables the bed to be thoroughly dried and aired and properly made. If extra help is needed in order to move the patient, a neighbour can generally be found who will come in daily at a fixed hour for a very small sum of money.

The majority of paralysed patients have fairly good appetites and ordinary diet can be given, but careful cooking and varied food are of great assistance to their general well-being.

### To Nurses.

We invite contributions from any of our readers, and shall be glad to pay for "Notes on News from the Nursing World," "Incidents in a Nurse's Life," or for articles describing nursing experiences at home or abroad dealing with any nursing question from an original point of view, according to length. The minimum payment is 5s. Contributions on topical subjects are specially welcome. Notices of appointments, letters, entertainments, presentations, and deaths are not paid for, but we are always glad to receive them. All rejected manuscripts are returned in due course, and all payments for manuscripts used are made as early as possible after the beginning of each quarter.

### Incidents in a Nurse's Life.

### A CASE IN THE MEDICAL WARD.

"Any new cases in to-night, nurse?" said the night sister as she signed the night chart. "One, I see, in No. 17. What is he—nothing very bad, I hope"?

"He seems very comfortable. Sister said he only came in quite late, and was not yet diagnosed; but she *thought* he was an hepatic case."

"Is he?" said sister with interest. "He seemed to be asleep as I passed. Ah! poor 8 has gone, I see, so you will have a light ward to-night. I hope you slept better to-day, nurse"? kindly scanning the pale face with shining, dauntless eyes.

"Oh, much better, thank you, sister"—gratefully. "Once that examination is over I know I shall be quite all right."

The night sister nodded sympathetically, as she knew well what the strain of massage and pharmacy entailed on a delicate, conscientious nurse, whose physical and nervous system was stretched almost to breaking-point by a long series of heavy cases and sleepless days. Then together they went softly through the long ward where twenty-six beds showed dimly in the flickering fire-light. The night nurse, in low tones, would give a quick, detailed report on each acute case, how the typhoid had had a relapse and gone back to milk diet; and then she would flash her hand-lamp on the four-hourly chart hanging on the wall that sister might see how the steady, "normal" line was sadly marred by a sudden upward sweep to 102 degrees. How No. 10 was "tapped" to-day; 12 had had a great deal of pain, but had been asleep since 9 p.m.; how 14 was not to have any more morphia; 15 had been put on fish diet; 17 was the new case, on milk only; 18-19 all well; 20 was so much better that they hoped he would be able to go to a convalescent home next week; and so on till the twenty-six beds were all visited.

It was late before the night sister finished the medical round. Two new cases admitted into "Mary," an operation in "John," and a birth in the maternity ward kept her so busy that she had no time to do more than give a "good day" to No. 17 in "Stephen"; but on the next morning she drew him out and ascertained that he was a lately-discharged soldier. He had spent the last five years in India, and always "enjoyed good health except for dysentery," and had come home a few months ago—then the old story, out of work; for weeks past he had had no regular employment, but earned an occasional shilling as "runner"; at last a cough and a "plaguey pain" forced him to seek medical advice, and so he found himself labelled No. 17, Stephen Ward.

"I shouldn't mind so much for myself," he said, "but there's the wife and the little 'un, and of course she frets, the missus do."

Sister looked keenly at the emaciated figure, with sunken eyes, and it seemed to her as if the man was visibly thinner and more shrunken since he came in. His harsh cough was more troublesome, his temperature showed a regular evening rise, and the night sweats seemed to have drained the strength out of him. Just then a probationer hurriedly announced, "Please, sister, will you come to 'Mary' at once," so further conversation was impossible; but before settling down again No. 17's hand stole under his pillow and produced a photograph of a young and pretty woman, at which he gazed long and tenderly before putting it back with a murmured "God bless you, my lass."

In the afternoon the visiting physician made his round, accompanied by the house physician, some twenty students, and the sister. No. 17 watched them as they slowly went round the ward, pausing longer at some cots than others. Sometimes screens were fetched and placed round the bed; why he did not know. Before long they reached his side, and he found himself surrounded by a group of doctors and students, and sister was close by with a clean towel over her arm. He could not follow the low-toned murmurs or remarks, nor understand why they tapped him and sounded him, and asked him so many questions; but just when he thought it was all over he distinctly heard the word needle. Sister bustled away to return with a white, three-cornered dish in her hand, someone noiselessly placed screens round his bed. "Lotion, please," said someone. "Thanks. Now, keep still, please. It's all over now," and then No. 17 became aware that his side had been punctured by a substantial-looking "needle."

"No pus, Warren?" asked the great man. "No, sir." "Ah," and he moved away to wash his hands. Someone then placed a bit of gauze and strapping over the hole made by the exploring needle, and after that was done the screens were removed, and he was left alone once more. When the night sister came round he was asleep, and so did not hear nurse tell her that "the diagnosis was not liver abscess, but pneumonia, and therefore they had moved his bed nearer the fire and given him a cotton wool jacket." No. 17 thought, when he awoke, that his hearing was not so good as it was, nor could he see so well. Was that really his wife—his Mary? How long had she been sitting there?—what time was it?—three o'clock in the afternoon? Why he must get up at once; it was time for parade. Where was his rifle? who had taken his belt? And so he rambled on while cold dismay gripped the heart of his little wife, who had been summoned by telegram some hours before. No. 17 lapsed again into a heavy stupor, from which he only slightly emerged when fed at regular intervals. He did not know his wife, who, poor soul, spent long hours by his side praying silently for only one look of recognition from those dull, lustreless eyes. The hours merged into days, and still no sign, no improvement; sometimes he was so still she thought he had passed away; sometimes in a short burst of frenzy or delirium he would call her name, but ever failed to recognize her voice or pleading touch.

What would be the end? She eagerly sought for hopeful looks or words from the doctor, sister, and nurses, but though they all tried to speak cheerfully she was not deceived. Slowly resignation forced itself on her; she could only pray his end might be peace.

At last one afternoon the visiting physician came again. Sister made a sign to her to withdraw, and the students clustered round the bed, hiding everything from her sight. A sort of mist came over her, she felt like screaming, and mechanically moved to the window for air, wondering vaguely if she were going to faint. Suddenly she heard sister's voice beside her saying: "I want you come into my room and sit down," and then, "Doctor W—— finds your husband very ill and it is absolutely necessary to perform an operation at once. I am sure you see how right he is to leave nothing undone for his recovery?" She nodded her head, only catching the import that an operation, though terrible, might mean recovery and life, and sister went on: "Try and not give way, but rest here quietly, and when it is over you shall go back and sit with him." Then gently she placed the shaking figure into an armchair, took up a small brass tray, on which were ranged four small glass bottles and a little leather case, and softly closed the door behind her.

The minutes passed by, a trampling of many feet was heard, a murmur of voices, the clashing of metallic instruments, silence, then more sound of footsteps. A nurse brought her in a cup of tea and bread and butter, and kindly forced her to eat and drink. She longed to ask for news, but somehow dared not, and nurse avoided the subject. Again she was left alone, but in a few minutes sister entered, and one look at her grave face told her what she dreaded to hear spoken. "They've killed him; I know they have," she sobbed. "Oh! my God, why did they not leave him to die in peace." She covered her face with her hands and rocked to and fro in an agony of tears. Sister stooped down and put her hand on her shoulder. "Listen," she said, "your husband is dead, but the operation was not performed; he died before it was begun, quite quietly."

### Simple Fracture of the Right Arm.

### EXAMINATION QUESTIONS FOR NURSES.

The question was as follows :—

How would you proceed to remove the coat and under garments from a patient who had a simple fracture of the right arm; and when the arm was sufficiently well to admit of a sleeve being used how would you re-dress him?

### First Prize.

If the patient was not in a very collapsed condition I should keep him in a sitting up position while the undressing was taking place, as it is much easier to remove the garments in this way.

I should endeavour as little as possible to cut the clothing worn by the patient, as this means a great deal to some people in poor circumstances; it is absolutely unnecessary to open the seam of the coat sleeve at all. I should get some one to support the fractured arm and I should then carefully withdraw the coat sleeve of the left arm first. The under garments I should ease well round the body, remembering always to withdraw the sleeves of the left arm first, then pass the garment over the head and down the affected arm last of all; if the garments are fully big this can be done without giving much pain. Should the under garments be woven and fitting tightly to the body, they should be carefully opened at the seam of the sleeve and across the shoulder if possible to give ease. This can easily be sewn up afterwards without showing any defects.

In dressing the patient I should always put the affected arm in the sleeve first, in this case the right arm.

"Belinda."

### Second Prize.

I should remove the coat from a patient who had simple fracture of the right arm, first by drawing the sleeve from his left arm, then passing the coat carefully behind him I should proceed to remove it from the right arm as follows: Passing my right hand up between the cuff of the coat and the shirt sleeve, I should support the fractured arm by letting it rest on mine, while I drew with my left hand the coat sleeve down as far as I could, I should then insert my left hand in the top of the sleeve, and slipping it in the place of my right hand, which I should carefully withdraw, and with it the coat.

I should draw the sleeves of the under-garments from the left arm first, and passing these over the patient's head, I should remove them from his right arm in the same manner as I had proceeded to do with the coat; always supporting the fractured arm as much as possible.

In re-dressing the patient I should pass the under garments over his head, and drawing right arm-holes well in front, I should carefully pass them over right arm first. The same precautions are to be taken when replacing the coat; always passing the sleeve over the right arm first. "Sidla."

### The Prize Winners.

"Belinda," a very painstaking competitor for some time, gains the first prize with a clear and concise answer. She notes the advisability of having the patient in a sitting position. A broken arm does not often cause collapse, and the difficulties of undressing a patient (with an injury to the arm) in a recumbent position is well known to all nurses of experience. She is also wise and thoughtful in deprecating the immediate slitting of garments. Poor people can ill afford any injury to their clothes if by a little management it can be avoided. Naturally, in a serious case, clothes must not be allowed to endanger health or life, but a clever nurse will usually succeed without meddling with the coat, and to unrip a vest is not a very serious matter.

"Sidla," who gains the second prize, sends a very good paper, but it would be impossible to insert a stiff and painful arm into a vest or shirt which had been already placed over his head. The arm must be put in first and the sleeve well pulled up to the shoulder, and even then I am doubtful if a woven vest could be managed further without slitting up the left seam or straight down the front.

Her other arrangements are very good and show thought and probably some experience.

### Honourable Mention.

This is gained by "Sassenach," "Dorothy," "Surrey," and "Chopin."

### Correction.

With a broken arm it is best *almost* invariably to rip the *outer* and not the inner seam of the coat, supposing such a measure to be necessary. A very large proportion of the candidates advice cutting the inner seam. This is wrong: consider the extreme difficulty of reaching that part of the sleeve, especially if the arm is flexed, whereas by opening the outer seam—easily manipulated—you can with a few inches more come to the shoulder seam and open that too if the case is a bad one.

### Question for December.

Imagine yourself called upon to nurse a fractured leg (Tibia and Fibula) in a poor outlying cottage where the bed was flock on a worn and loosely stretched sacking. What arrangements should you make to keep the leg in good position? Remember that appliances and money are absent, and shops, even, many miles distant! The Examiner.

### Rules.

The competition is open to all. Answers must not exceed 500 words, and must be written on one side of the paper only, without divisions, head lines, or marginal notes. The pseudonym, as well as the proper name and address, must be written on the *same* paper, and *not on a separate sheet*. Papers may be sent in for 15 days only from the day of the publication of the question. All illustrations strictly prohibited. Failure to comply with these rules will disqualify the candidate for competition. Prizes will be awarded for the best two answers. Papers to be sent to "The Editor," with "Examination" written on the left-hand corner of the envelope.

In addition to two prizes honourable mention cards will be awarded to those who have sent in exceptionally good papers.

N.B.—The decision of the Examiner is final, and no correspondence on the subject can be entertained.

Any competitor having gained three prizes within the current year shall be disqualified from taking another until 12 months shall have expired since the first prize was gained.

### Death in our Ranks.

There passed away on Saturday, November 18, at Southport, after three years of weary suffering and helplessness, Nursing Sister Mildred Armytage Richards, of the Army Nursing Service Reserve, who was invalided home from South Africa in August 1902, after two years' service during the war. She was trained at St. Mary's Hospital, Paddington, and afterwards held the post of assistant matron at the South Western Hospital, Stockwell; from Stockwell she went to Egypt to take up private nursing, and was ordered to South Africa in May 1900. She will be much missed by her many friends, to whom she was endeared by her gentleness, fortitude, and bright, loving disposition.

### Central Midwives Board.

A meeting of the Central Midwives Board was held on Thursday, November 23, at which the following members were present: Dr. Champneys (Chairman), Mr. Ward Cousins, Dr. Dakin, Mr. Fordham, Mrs. Latter, Miss R. Paget, Sir William Sinclair, Miss Wilson, and Mr. Parker Young.

After the minutes of the last meeting had been confirmed, the first business considered was a letter from the clerk of the Strood Rural District Council, asking the opinion of the Board as to the right of a Local Supervising Authority to inspect a midwife not resident within their jurisdiction, but from whom they have received notice of intention to practise within their district. A similar letter from the Alton District Council was also read. After some discussion as to whether residence or practice placed a woman under the jurisdiction of a supervising authority, reference was made to Section 8 of the Act, from which it was clear that the latter was the qualification considered, and the Secretary was accordingly requested to point out to the correspondents the wording of the Act. Several members of the Board remarked that these were examples of how difficulties arose through delegation of powers to Rural District Councils.

### The Examination System.

A letter from Dr. Fothergill, on behalf of the examiners for the Manchester Centre, conveying certain suggestions as to the examination-system of the Board, was then considered. The chief suggestions were that the examination papers were too easy to afford any satisfactory test, as was proved by the number of women who passed the written, but were unsuccessful at the oral examination; that an attempt be made to acquire some uniformity of training; that an equal number of examinations be held in London and in the provinces; and that three months elapse before any candidate, if unsuccessful, be allowed to offer herself for examination a second time. After some discussion Mr. Ward Cousins moved and Sir William Sinclair seconded that these proposals be circulated to the members of the Board and be placed on the Agenda for the next meeting. This was accordingly resolved.

It was decided to consult the Registrar-General on a letter from the Clerk to the West Riding Sanitary Committee asking the Board to furnish the Registrars of Deaths within the area of the West Riding with annual copies of the Midwives Roll, in order to facilitate the notification to the Board of the death of any midwife, as required by Section 8 (6) of the Midwives Act.

The Secretary was asked to express the inability of the Board to comply with the request made in a letter from the Secretary of the Cheltenham District Nursing Association, asking the Board to extend the time for signing the schedules for the February examination in the case of the four nurses now being trained by the Association.

The Board had so many such requests that its only course was to refuse them all.

The Secretary reported that at the October examination there were 471 candidates (eight of whom did not appear) as against 311 in June; the percentage of failures over all candidates was 24 as against 22.8 in June. The number of candidates in London alone was 346, and the percentage of failures 23.7. The profit on the examination was £52 as against a loss of £1 at the last examination, which was due to the increase in the number of candidates.

### The Business of the Board.

The report of the Committee on the business of the Board was brought forward and its recommendation that the Standing Committee be held on the Thursday before the Board meeting was considered. Sir William Sinclair strongly protested, saying that it was too much to demand from the country members that they should give two days to the Board's business a month, sometimes, indeed, three, when there was an appeal-case meeting in addition. He suggested an all-day sitting. Mr. Parker Young said that the Committee's report was really based on the answers given by members of the Board to a circular sent round by the Committee: their only wish was to expedite business. Dr. Champneys said that their desire was to relieve the country members. Sir William Sinclair said he thought that a greater number of the technical points might be left to the Chairman and Secretary to decide, and that the Standing Committee might then be abolished. Finally, it was decided to bring up the matter at the January meeting. The date of the next meeting was fixed for December 14.

### Applications Refused.

The report of the Standing Committee of November 9 was then considered. The application of Dr. J. H. Bellamy, of the Union Infirmary, Firvale, Sheffield, to be recognised as a teacher, had been reconsidered and was recommended for approval. The Board accepted the recommendation. The Eastern District Hospital and the Western District Hospital, Glasgow, and the Chorlton Union Hospital, Manchester, were recommended for approval as training schools. Sir William Sinclair opposed the recommendations on the ground that, with regard to the two former, they were not required, since the work of training was splendidly organised at other institutions in Glasgow; that with regard to the latter the hospital was structurally unfit and the arrangements bad. The Board had already approved of these institutions subject to the suitability of their syllabus, and Sir William's opposition met with no support. Fulham, Greenwich Union, and Kensington Union Infirmaries were "not approved till defects were remedied." Mr. Parker Young urged that a distinction should be made between Kensington and the others, since the defects at Kensington were not structural, the large number of cases of ophthalmia at Kensington being the chief objection to its approval: he would like Kensington to be "approved, subject to the defects being remedied." Mrs. Latter seconded the proposal, but the motion was lost.

With regard to the approval of applications as teachers under Rule C. I. (3) Sir William Sinclair moved that all those applicants unattached to public institutions be not approved. He felt that some doctors wished to improve their own status in small villages by forming classes and becoming recognised teachers. He wished the same principle to be enforced with respect to the applications of certified midwives under Rule C. I. (3). His proposals were not seconded, and the recommendations of the Committee were adopted.

Mr. Parker Young moved

That £5,000 now on deposit be invested in a Trustee Security, in the names of Dr. Champneys, Sir W. Sinclair, and Dr. Dakin.

Sir William requested that his name be omitted. Dr. Champneys and Dr. Dakin were accordingly appointed trustees. Mr. Parker Young moved that the investment ordered by the previous resolution be made in India 3 per cent. stock. This was carried.

### Wants and Workers.

Would any lady kindly help a district nurse by sending some left-off underclothing, or any useful garment to her, for her district? Address Nurse Anderson, District Nurse, Porlock, Taunton.

### Resignations at the Queen's Jubilee Hospital.

We received on Monday an intimation from Miss C. Moor, Matron of the Queen's Jubilee Hospital, Earl's Court, that the sister, staff nurse, and herself had resigned, and would sever their connection with the hospital forthwith. Our representative saw Miss Moor on Tuesday, and she made the following statement:—

"When, last February, Miss Mackintosh, who was at that time Matron of the Queen's Jubilee Hospital, resigned, Miss Moor, who had acted as temporary matron for six weeks in the summer of 1904, was asked to return there, on the distinct understanding that this time the post would be a permanent one. Miss Moor, on this condition, accepted the post. At a meeting of the Board about three weeks after Miss Moor took up her duties, she was called in and informed that as the Board had got into trouble with the authorities of King Edward's Hospital Fund for filling up the vacancies in the hospital so quickly, without advertising them, they were unable to give her the appointment as matron for three months; but in the minds of the Board she was appointed. Miss Moor replied that she had given up good work to fill this post, and the understanding had been that it was to be permanent.

Miss Moor states that the hospital, when she went into it, was in a very dirty condition. The Nurses' Home, where seven or eight nurses were accommodated, had a charwoman for two half-days in the week as the only attendant. The night and day nurses slept in the same room. When the visitors of King Edward's Hospital Fund made their inquiry, Miss Moor was asked by the Board to state, in reply to any questions as to whether her post was permanent or temporary, that the post had not yet been advertised. The Visitors, on the occasion of their annual inspection, personally complimented the matron on the condition of the hospital.

In October, after one of the meetings of the Board, Miss Moor was summoned before the chairman and one of the senior surgeons, and was informed that they had decided to offer her the post of matron for three months. On Miss Moor replying that she had understood the post to be permanent, the Chairman agreed with this view, but stated that as she had had no previous training as matron, they had unanimously decided to advertise the post, and if she would send in her application it would be considered with the rest. If this offer were not accepted they must advertise the post at once. They stated that they had no fault to find with her and at the end of three months would give her the best testimonials. The surgeon also suggested that if Miss Moor cared to take the post of housekeeper she could have it at once as a permanency. The next day Miss Moor learnt from other members of the medical staff that they had heard nothing of the proposed step and had repeatedly urged her appointment as matron upon the Board. Miss Moor then wrote a letter to the Board complaining of the treatment she had received. Both the matron and sister complained that their authority over the nurses had been undermined by a member of the staff, who had informed one of the nurses that if anything went wrong they were to report to him. A nursing committee was formed, having one of the surgeons as Chairman, and when the sister complained of a probationer whom she had reprimanded, "reporting" her to this gentleman, the nurses were instructed in future to write a letter to the Chairman of the Nursing Committee.

Miss Moor, who was trained at the Homœopathic Hospital, where she remained for four and a half years, has had eight years' experience. The sister, who was trained at Guy's Hospital, has had eight years' experience. As far as Miss Moor can gather, the only reasons alleged against her appointment are that she has a certificate from the Homœopathic Hospital and is a Roman Catholic."

### Glasgow Matrons and State Registration.

A meeting was convened by Mrs. Strong, matron of the Royal Infirmary, Glasgow, and held in that institution on Thursday last week, in order to discuss the question of State registration for nurses. There were present Lady Chisholme and Mrs. James A. Napier, lady managers, and the following matrons: Miss Shannon, Western Infirmary; Miss McFarlan, Victoria Infirmary; Miss Husband, Maternity Hospital; Miss Adams, Ruchill; Miss Wright, Stobhill; Miss Marchant, Eastern; Miss Mosely, Oakbank; Miss Laudles, Knightswood; Miss Scott, Schaw Home; Miss Berwick, Sick Poor and Private Nursing; Miss Whitecross, Incurable; Miss Wilson, Lock Hospital; Miss Torrance, Cancer Hospital; Miss Grant, Elder Hospital; Miss Wright, Private Hospital for Women; Miss McEachran, Woodside Terrace Home; Misses Waddington and Aitchison, Sandyford Home; Miss Brown, Regent Home; Miss Melrose, assistant matron, Royal Infirmary; and the lady superintendents of the various departments of that institution.

Apologies for absence were sent by Miss Alexander, Paisley Infirmary; Miss Simpson, Sick Children's Hospital; Miss Chalmers, Eye Infirmary; Miss Tisdall, Queen's Crescent Home; and Mrs. Sinclair, Belvidere Hospital. Mrs. Sinclair's absence was much regretted, as she was one of the pioneers in nursing reform in Glasgow, and has devoted the past forty years of her life to the bettering of the condition of nursing, and is always foremost in any movement tending to its advancement.

The meeting, which was quite informal, was opened by Mrs. Strong remarking that her reason for asking the matrons of the Glasgow hospitals to meet was that she was now a member of the Bill Committee for the proposed State registration of nurses, and wished to ascertain the opinion of her fellow-matrons on the subject. She further stated that her opinions had not changed; they were the same as given to the public in 1895 and again in 1901. Mrs. Strong then asked for the opinion of the meeting in regard to the State fixing a nurses' curriculum and determining examination. There was an almost unanimous finding that this is desirable. Some interesting points were raised; one, upon the position of special hospitals, by Miss Torrance, of the Cancer Hospital; and another by Miss Aitchison, Sandyford Home, in regard to small hospitals in general.

The meeting terminated by Miss Husband proposing a vote of thanks to Mrs. Strong for the able and clear manner in which she had put the subject before them.

### Prize Distribution at Bristol General Hospital.

On Thursday last week the prizes to successful probationers at Bristol General Hospital were distributed by Mr. J. Storrs Fry, Chairman, and after the ceremony an "At Home" was given, at which over forty guests were present, consisting of members of the staff, committee, and their friends. The prizes were awarded as follows: Gold.

medal, Nurse Hadley; silver medal, Nurse Brill; certificates of merit, Nurses Davies, Jenkins, Lewis, Philo, and Skinner. Anatomy : 2nd year (1st prize), Nurse Grandin, (2nd prize) Nurse Walmsley; 1st year (1st prize), Nurse Grahame, (2nd prize) Nurses Jordan, Pontifex, and M. Davies. Physiology : 2nd year (1st prize), Nurse Grandin, (2nd prize) Nurses F. Keen and Armstrong; 1st year (1st prize), Nurse Grahame, (2nd prize) Nurses Pontifex and M. Davies. Surgical nursing : 2nd year (1st prize), Nurse Gradisky, (2nd prize) Nurses Walmsley and Gauntlett; 1st year (1st prize), Nurse Perry, (2nd prize) Nurses Webb, Grahame, and Sampson. Medical nursing : 2nd year (1st prize), Nurse Harvey, (2nd prize) Nurse Grandin; 1st year (1st prize), Nurse Pontifex, (2nd prize) Nurse G. Keen.

### Everybody's Opinion.

[Correspondence on all subjects is invited, but we cannot in any way be responsible for the opinions expressed by our correspondents. No communication can be entertained if the name and address of the correspondent are not given as a guarantee of good faith, but not necessarily for publication. All correspondents should write on one side of the paper only.]

### CHRISTMAS NOT ALWAYS A JOY.

The Matron of Taunton and Somerset Hospital, Taunton, writes : I most cordially sympathise with you in your endeavour to put an end to the Christmas taxation of sisters and nurses which is prevalent in so many hospitals. I am happy to say that the custom has not been observed in this hospital since my first Christmas, in 1900, when I refused to accept any presents from the nursing or domestic staff, and I have further succeeded in abolishing the system of testimonials every time one of the sisters was leaving. I think I may safely say that there exists a very true fellowship of the right kind between matron, sisters, and nurses here, in spite of these restrictions. With regard to Christmas decorations and entertainments I have but followed in the footsteps of my predecessors in sending out an appeal at the beginning of December to all friends of the hospital in the neighbourhood, and the result has always been an ample response both as regards money and gifts, so that we are free from all pecuniary anxiety. The chairman, the medical staff, and other members of committee are always generous contributors to my fund.

"M. R. N. P. F." writes : As I was the first to start the subject of "Christmas presents" in The Hospital, I should like to thank you very much for taking the matter up so thoroughly, and especially for the article in this week's issue. It is a question in which the matrons ought to interest themselves; but I am afraid most of them will not do so. The sisters at this hospital have referred the matter to our matron, but unfortunately not altogether successfully, though she does suggest the use of boxes as one of your correspondents did. This, I think, savours too much of "sending the hat round"! Possibly you might like a few facts from my own experience during a period of ten years in hospital. I have been in good provincial hospitals and in a large children's hospital, where one naturally does a great deal of decoration, etc. In the latter case no money was provided by the committee, so that it cost me usually about £7, inclusive of gifts to the staff. Both my wards were large, and contained thirty-two cots and several cradles. At another hospital, where I went to do temporary duty as surgical sister, no decorations, such as flags, lights, etc., were in stock from former Christmases, and although I had only been there six weeks I found I had to decorate largely, besides giving the usual presents, and then I was told that I must provide a concert in the ward. I remonstrated with the matron, saying that I knew no one in the place, and could not afford it; short of absolutely insisting on it she let me see that it would be considered very extraordinary if the usual routine were not carried out, and by great efforts I managed to find musicians, who gave their services free certainly, but expected refreshments afterwards. These also were provided by me, and I was responsible for the hire of a piano. At a large hospital of 400 beds, where I spent some years as sister, I usually found Christmas could not be passed without disbursing about £6 of £7, though the committee provided £1. While we were very grateful for this, it barely covered the flower and plant expenses for large wards, these being so very dear at Christmas. If only matrons would put their foot down and decline to receive valuable presents themselves possibly the present state of things might be altered. I know of one lady, a relation of my own, who has been in the same hospital for about fifteen years, and her sitting-room is almost furnished with most lovely chairs, tables, writing-desk with sliding top, etc., all subscribed for by the nursing staff. However one may respect and love one's matron and enjoy choosing and giving some little token of affection, this wholesale taxation cannot fail to make Christmas a "weariness to the flesh for some members of the staff."

### A QUESTION OF PROCEDURE.

"Matron of a Nursing Association" writes : May I trouble you with the following question? I was asked by a lady the other day to send a visiting nurse to a patient. The nurse reported to me that there was no doctor in attendance, but that she was requested to apply an ointment prescribed by a lady who gives free advice but who charges 35s. for each box of ointment. The patient's mother told the nurse that she wished her first to sponge down the back with acetic acid and then rub in the ointment over the spine. I wrote to the lady explaining that I could not take the responsibility of sending a nurse where there was no qualified medical man or woman in attendance. In talking the matter over with one of our directors, she said she did not see it from my point of view, thought the responsibility rested with the patient and her mother, but at the same time said I had better do what I considered to be right. Should I have been acting correctly in taking up the case?

[We think that you were perfectly right in declining to allow the nurse to attend the case.—Ed. The Hospital.]

### DISTRICT NURSES AND MATERNITY WORK.

"Fair Play" writes : As one of those despised nurses who combine general and maternity work, I wish to thank "One Interested" for her letter; with which I entirely agree. I also wish to inform "Puzzled" that a nurse would not "dress all kinds of wounds" when attending a maternity case. She would exercise a little common sense. How would "Puzzled" herself act if she were nursing in district a case of recent operation? I have worked in my present district eleven years, and although not a poor one, I am sure that it could not maintain two nurses. I grant that the life is sometimes hard, and one of compulsory self-denial. But why—I ask with all deference—should the combined work be more impossible for a nurse than for a doctor, who may have a large general and midwifery practice, and is, besides, liable at any time to be called to infectious cases, or perhaps to a post-mortem?

### IS THERE ANY ADVANTAGE IN JOINING THE PENSION FUND?

Miss J. Anstey, late matron of Bolton Infirmary, writes : Nearly fifteen years ago I took out a policy for a pension, and also one for a pound a week in sickness, in the Royal National Pension Fund for Nurses. I was quite strong at the time, and continued so for between six and seven years, when, through illness, I was compelled to give up a good appointment. For more than seven years I have, on and off, been receiving one pound a week. Sometimes I have been a little better, and able to visit my friends, but never able to take up work. As I look back to the day I joined I can only exclaim, "I made a good investment." I would like to mention two facts. First, I have paid in for sick policy premiums to the amount of twenty-five pounds thirteen shillings; secondly, I have received in sick pay between two and three hundred pounds, and I am at the present time receiving one pound a week. Here I must add another fact. Although living in a provincial town, I have from time to time gone up to see the medical referee free of expense. The courtesy, kindness, skill, and careful consideration shown to me both by doctor, secretary, and all the officials have been such as to demand my grateful appreciation. I cannot look forward to working again, but I can look forward to a comfortable old age when in 1908 I enter upon my pension, and trust that many nurses will find the sick fund as valuable as I have done. Why am I writing this? In order to encourage all nurses to join this most excellent fund and provide for themselves should sickness or an incurable disease overtake them, as in my case. A year ago I was speaking to a group of nurses, and one of them replied, "Look at the money you have to pay for sickness premiums; it is all lost money." Has it been my experience? Let the above facts give the reply to that question.

### THE RURAL POOR AND THE MIDWIVES ACT.

"A Friend of Midwives" writes: The number of women sent out through the organisation of the Rural Midwives Association to work in the rural villages as midwives and nurses amongst the poor—women of the working class who are ready to give practical help and sympathy to the cottage mother—is continually increasing; and the recognition of its value from doctors and nursing associations in the fifteen counties where it already has supplied fifty-seven midwives is distinctly encouraging to the promoters. The financial department is so well organised that the actual training is in great measure self-paying, but—as in all work of this sort—a margin of necessary expense must be met by voluntary assistance. The demand for another £150 a year to meet current expenses is, all things considered, a modest one, and this addition to the funds would be a great relief to those who so willingly give their time to the work. These rural midwives enable the mother to rest in peace and regain her strength without anxiety as to her household needs; they see that the baby has a fair start in life, with the nourishment suited to its condition, and the mother is taught how to manage and feed it when the midwife has gone. Many of the women and children now treated in hospitals and convalescent homes would not need to become patients if throughout the land, in every village, these valued friends of the rural poor could be established. There is in connection with the Association now a training home in Bermondsey, which after six months' work is already becoming so popular among the poor that as much as £5 in a month is paid by them towards the services of the midwife and her probationers. For this no help is asked; but a few free tickets for the very poor who cannot afford even the moderate sum charged—£1 1s.—will do all that is required for three poor women, and tend and nurse each and the babe until convalescent. The Secretary of the Rural Midwives Association, 47 Victoria Street, S.W., will, I am sure, be glad to give all information about this and the general work.

### BREAD CAST UPON THE WATERS.

"An Edinburgh Nurse" writes: District nursing is very trying sometimes, especially in large cities like Edinburgh. Often when on our daily rounds we feel sad, and get depressed, seeing so much sickness and poverty. We come away saying, "What can we do?" feeling helpless in ourselves, and longing for a little sympathy or word of encouragement. May I tell you about a little girl, whose mother I have lately been visiting in a very comfortless home. The little girl is ten years of age, and her name is Mary. She gets up in the morning, makes the breakfast, tidies the house before going to school. After school hours she goes to the house where her mother, when well, does charing, and here Mary has been trying to take her mother's place, cleaning the doorstep, running messages, and making herself useful in many little ways, at the end of the week getting a few coppers, which she gladly gives to her poor mother. In the evenings Mary would ask her mother if the nurse had been to-day, and one day she said, "Mother, I do want to see the nurse. She must be nice. When Saturday comes, if I get my work done in time, will you let me watch for nurse coming?" When Saturday morning came, and I got to this home and saw the little girl sitting, and was told the story, how Mary had been up early getting the work done so that she might be able to see me, whom she had pictured in her mind as nice, it made me feel vexed with myself for ever getting depressed, and I felt that I should like to tell my sister nurses who may, like myself, feel the work trying at times, not to lose heart. I may add that Mary came to see me last Saturday with a bunch of flowers, showing how grateful she was for the little done to her mother.

### "PRESENTED AT COURT."

"A District Nurse" writes: To be presented at Court is the "summum bonum" of happiness to some people. My experience was not this. I have always been a law-abiding citizen, and kept out of the arm of the law, but cycling home late one night my lamp went out. Too tired to get off and re-light it, I rode on. It was moonlight, and I was thinking how beautiful the country looked all around when my thoughts were rudely disturbed by a voice demanding to "know where my light was." I dismounted and explained to P.C. Bumble that my lamp had gone out. I re-lit it and rode on, expecting that the matter had ended, but, alas! short lived were my hopes, for in due course a blue paper was served upon me. So zealous in the discharge of his duty had the constable been that he had even invented my Christian name! My feelings can be better imagined than described as I cycled in to appear at the local police court. I had visions of a judge in wig and gown, and of being asked if I were "guilty or not guilty." A small room, with scarcely breathing space, and a few grim veterans sitting to execute justice, a sprinkling of "drunks and incapables," belonging to the army of the unwashen, met my gaze. I was accommodated with a front bench beside "Betsy Prig" of the local paper. The charge against me was read out. P.C. Bumble went into the witness box, and, as usual, exaggerated the distance I rode. I knew that for every word I said I should have to pay dearly, so, like Uriah Heep, I decided to be "very 'umble." In fear and trembling I stood, the magistrate grunted to the effect "that I might have walked had my lamp gone out." Fine 10s. I left that Court a sadder and a wiser woman.

### Appointments.

[No charge is made for announcements under this head, and we are always glad to receive and publish appointments. The information, to insure accuracy, should be sent from the nurses themselves, and we cannot undertake to correct official announcements which may happen to be inaccurate. It is essential that in all cases the school of training should be given.]

Aberdeen Eye Institution.—Miss Annie B. Boyd has been appointed matron. She was trained at Plymouth Royal Eye Infirmary, and has also had experience in general work at Princess Alice Hospital, Eastbourne, and Dr. Savage's Private Hospital, Birmingham. She has since been matron at the Bath Eye Infirmary and assistant matron at the Nurses' Institution, Chelmsford.

Croydon Union Infirmary.—Miss Marie Andrews has been appointed night superintendent. She was trained at Kensington Union Infirmary, where she was afterwards charge nurse; she has since been charge nurse at Hammersmith Union Infirmary and ward sister at Croydon Union Infirmary.

Gloucester General Infirmary.—Miss D. A. Copley has been appointed Home Sister. She was trained at the General District Infirmary, Ashton-under-Lyne. She was afterwards private nurse for the Oldham Nursing Association, has taken temporary matron's duty at the Gorleston Cottage Hospital, and has been ward sister at the Town's Hospital, and at the Western District Hospital, Glasgow.

Institution of Trained Nurses, Leicester.—Miss Eileen F. Beamish has been appointed lady superintendent. She was trained at the Manchester Chest and Throat Hospital, and at the Bradford Royal Infirmary, where she was gold medallist of her year and sister of the children's and male medical wards. She has since been attached to the Yorkshire Co-operation for Nurses and Nursing Homes, Leeds, and sister-in-charge of the male blocks of the National Consumption Hospital, Newcastle, County Wicklow.

Jessop Hospital for Women, Sheffield.—Miss Louise Marshall has been appointed staff nurse. She was trained at Derbyshire Royal Infirmary, and has since been charge nurse at Penistone Isolation Hospital, near Sheffield.

Leeds Maternity Home.—Miss Edwards has been appointed matron. She was trained at Salop Infirmary, Shrewsbury, and for maternity at Clapham Maternity Hospital. She has since been sister at the Salop Infirmary and at the London Hospital.

Meath Home of Comfort for Epileptic Women and Girls, Godalming.—Miss Leigh Clare has been appointed Lady Superintendent.

North Evington Poor-law Infirmary.—Miss Mabel E. Bromfield, Miss Elizabeth Brown, Miss T. M. Dodge, Miss E. A. Gilbert, Miss Matilda Gray, Miss Ellen Farman, Miss E. Johnson, Miss Edith Robinson, Miss Ada Strivens, Miss Harriett Gimson, Miss E. Weaver, and Miss Maud Vokes have been appointed staff nurses. Miss Bromfield was trained at Stapleton Union Infirmary, Bristol. Miss Brown was trained at Reading Union Infirmary. Miss Dodge was trained at Isleworth Union Infirmary, Brentford. Miss Gilbert was trained at Holborn Union Infirmary, where she has since been staff nurse; she has also done private nursing. Miss Gray was trained at Birmingham Union Infirmary, and has been nurse at Leek Union Infirmary. Miss Farman was trained at Tonbridge Union Infirmary. Miss Johnson was trained at St. Mary (Islington) Union Infirmary, and has since been staff nurse at the North-Eastern Hospital, Tottenham. Miss Robinson was trained at Barton-upon-Irwell Union Infirmary, and has since been staff nurse at Eastry Union Infirmary, Dover. Miss Strivens was trained at Rochdale Union Infirmary. Miss Gimson was trained at Crumpsall Union Infirmary, Manchester. Miss Weaver was trained at Holborn Union Infirmary, where she has since been staff nurse; she has also been nurse at the County Hospital Newport, Mon. Miss Vokes was trained at Mile End Union Infirmary, and has since been staff nurse at Camberwell Union Infirmary and the New Women's Hospital, Euston Road, London.

### Presentations.

Leicester Institution of Trained Nurses.—Miss J. M. McHardy, Lady Superintendent of the Nurses' Institution, Leicester, who has been appointed Lady Superintendent of the Nurses' Institution and Nursing Home, Clifton, Bristol, was on Friday last presented by the Committee with an illuminated address and a silver tea-service and hot-water jug. The presentation was made by Mr. S. F. Stone, Chairman of the Committee. The nursing staff also presented Miss McHardy with an inlaid harewood and silver tea-tray and silver trinket-box. She has held the post of Superintendent for eight and a half years, and her departure is generally regretted.

Matteshall, East Dereham.—Miss E. Carless, who for the last four years has acted as midwife in Matteshall, and has just been appointed head nurse at Battle Union Infirmary, has been presented with a dressing-case, with silver fittings, from patients and friends in the village.

### New Books for Nurses.

Golden Rules of Sick Nursing. By Dr. W. B. Drummond. (Bristol : John Wright and Co. Price .)

This excellent little book is not one for the library, but it might well find a place in the pocket of every probationer. From the first page to the last it is full of sound teaching on the nursing of the every-day cases that any intelligent beginner will want to have explained to her; and all the directions are given in the simplest language. The single page which explains "how to pour out a medicine" would, if taken to heart, save the sister of a ward many wearisome repetitions. It is a pity that the author has not included any hints as to the preparation of a patient for an anæsthetic; and it might be objected that a blanket wrung out of water at a temperature of 110° would be more chilly by the time it reached the patient than would be suitable for a warm pack; but the instructions given are, in the main, "Golden Rules."

The Nursing of Sick Children. By Dr. Burnet, of the Royal Hospital for Sick Children, Edinburgh. (London : Scientific Press. 1s. net.)

The eleven lectures which make up this little book give clear and useful information on children's ailments. The first lecture is concerned with those general points which must be remembered as differing from the practices in adult nursing. It mentions the irregular pulse often met with in quite healthy infants during sleep, the rapid respiration which is best counted by watching the movement of the nostrils, and the various ways of getting a dose of medicine down. The chapter on feeding is a very good one, though it is to be noticed that the author prefers milk, cream, and egg to the more usual rectal feed of albumen and starch. He lays necessary stress on "the avoidance of over-feeding and in seeing that regularity is observed. As a rule an interval of three hours between each meal is the shortest that should be allowed, and between times abundance of water should be supplied whether the child asks for it or not." And again later on : "If the food is vomited shortly after it is given, this generally means that too much has been fed to the child, or that it has been fed too rapidly." In the chapter on cases of diarrhœa and vomiting a needful warning is given as to the infectiousness of the stools and the means to be taken to render them harmless. The author advocates washing out the bowel in these cases with a weak solution of boracic acid lotion, which he prefers to the administration of castor oil. The management of children during an attack of acute rheumatism and during convalescence occupies the next lecture. The instructions for nursing children in convulsions or fits are worth careful attention. The writer says : "It is the usual custom to place every infant or child who has a fit into a bath of hot water. This we consider not only unnecessary, but positively harmful in the majority of instances. The hot bath is given with a view of relieving the congestion of the brain which is supposed to be present; but in our opinion it not infrequently serves to increase the congestion. Besides, a hot bath often excites the little patient, and tends to bring on quite a succession of convulsive seizures." The last chapter is one of the best in the book, consisting of many excellent hints on caring for the ailments of throat, nose, and ear.

### A Book and its Story.

### LITTLE LEGENDS.*

To open a book which, judged by its title, suggests an interrupted story, and to find instead a collection of short tales is always more or less annoying. "The Cloak of Friendship," Mr. Laurence Housman's latest book, comes under this head, and the title concerns one story only, which, like the six others that follow it, is a quaint compound of legend and ethics. "The Cloak of Friendship" seeks to enforce by allegory the duty of love to dumb animals. Children may be attracted by listening to it, for the story's sake, and children of a larger growth, having still the child's heart, may find a fascination in the truths underlying its fantasy. An old king lies dying in Finland, and as in all properly constructed stories of yore a triple choice is offered to his sons as a test of fitness to be ruler of the kingdom after him. His secret treasures are unfolded for their inspection, each having a mystic significance. The first is the sword of sharpness, whose force is so great that no enemy of the king can resist it. "It has won the wars," said the king. The second treasure is the cap of darkness, whose property is to render the wearer invisible. To the king it had given the doubtful joy of discerning friends and enemies, and of going unperceived among his people, and finding out any treacherous designs against his throne and State. Sadly he exclaims: "It has brought me to old age." The third treasure is the cloak of friendship, whose magic can only be exercised by actual contact with the person wearing it. It is as yet limited in its effect, and is unable to attach all persons to the wearer, being as yet in the making. But to the king it had brought some true friends. The king has two sons. Wisdom of the sorrow working order lies with the younger; the elder chooses the sword of sharpness, for to him strength and homage crowned by victory are promised by its possession. The younger son exclaims, on hearing his decision: "My brother's choice deprives me of nothing that I wish for. Let the sword be his. I choose the cloak of friendship." The king approves the choice, and when dying leaves the throne to the less belligerent son, who sits at home in peace, happy in the possession of the magic cloak, while from afar comes news of the victories of his brother. A quaint description of the cloak follows: "Now this cloak, which the king had told that it was yet amaking, but not made, was fashioned on this wise; of every animal in the world it contained one hair, and of every kind of bird under heaven it contained one feather; but it contained not the hair of a man. For whatever gave hair or feather to the weaving of that cloak must give life also and freely, knowing the cost beforehand. Therefore was the cloak woven from the covering of all living kinds under heaven, saving of man; for no man would give his life freely to let one hair of his head carry with it the virtue of self-sacrifice to the weaving of the cloak of friendship." Once the property of a great wizard, learned in the speech of all living creatures, it had come at his death into the king's possession. Going from one tribe to another he had learned from feather and fur which among them was ready to die that he might have part in the magic cloak. He answers their enquiries as to the reward awaiting the sacrifice thus: "It is a cloak that will bring those that have share in it to be in friendship and understanding with man." After they have heard this, from every tribe one was found ready to give up life for that friendship and understanding which have been lost since Adam and Eve were driven out of Eden. So the cloak came into making, but so far was not finished, as no man had been found to sacrifice his life for the completion of the cloak. Even the wizard, when he was dying, would not give one day of his life that the work might be accomplished. Nor the king himself. In dying he had given a parting injunction to his son: "Thou art full of life, but do not die yet," and laying the cloak of friendship on the boy's shoulders he passes out of life. Left in possession of the cloak, the young king feels the moving of a new spirit within him when wearing it which puts him in friendly fellowship with all living things. Their speech and their ways are no longer strange to him; possessed by the charity which knoweth all things he had the sympathy which regards all creatures with tenderness. The following illustrates the picturesque setting, the passage describing the result of the wisdom that has come to him: "Thus he knew that on Christmas night, when the ox and the ass are talking in stall, there are others out and seeking to get word, while Divine rumour runs between earth and the stars, and to learn what joy means of which the ox and the ass already know a little. But he knew also that it was not well to tell too much, else might the kind hearts of dumb things break with impatience for the relief which is surely one day to come, and with wonder at the hardness of their hearts. One Christmas night it was dark, though the ground was covered with snow; and the little king put on his cloak of feather and fur and stole out to the fields. And all round him in the air he felt the rumour of the Divine Birth moving to give rest to the hearts of men." The young king meets various animals who are losing their way or flying from the pursuit of another whose prey they would have become; all are arrested and drawn under the shelter of his cloak, where enmity dies, and they tarry in peace. Then the elder brother returns from his martial encounters, and the little king, persecuted and shunned by his people for his unconventional methods of dealing with his animal friends, is treated as a dealer in black magic, and finds his position untenable. He resigns the crown to his brother. Once more Christmas is near. The new king has made discord and war, in place of peace, in his own country. The dethroned king has only his cloak of friendship left to cheer him for the loss of his people's confidence. That kept his heart warm. . . . But often he would look at the frayed edge where it lacked finish, because no man would give his life that the bond of friendship between man and beast might be made whole, and would wonder if it could be right for a man with a soul that lives to give up life for the sake of the beasts that perish." On Christmas night he prepared a feast; and the ways of the city swarm with wild beasts going peaceably towards the feast that had been prepared without tables. The king is infuriated when he hears of this, and gives orders for his archers and huntsmen with their hounds to make ready for the destruction of the animals. "Quick, with sudden fear, the younger brother cried: 'Brother, you will not kill?' 'As I choose,' answered the other. 'Nay, for my honour is pledged to them! I have promised that they shall get no hurt. . . . If one of them is harmed I am dishonoured for ever.' 'Thy dishonour is in making promises thou canst not perform,' replies the king. 'Nay, brother, for what I have promised that I will perform.'" Then he craves the kiss of peace from his brother, "that afterwards it may be known that there was love, and not hate, between them upon this night, which is the joy of all Christian souls." Taking up the unfinished edge of the cloak of friendship and weaving into it one hair of his own head and stretching it wide he draws it around his brother; "and so standing in bond to his brother he saw his face change and grow merciful and knew that his gift had been granted, so that he need fear no longer for the lives of the guests he had brought in with him that night." "The Cloak of Friendship" is, to our mind, the best in this collection of allegorical legends.

* "The Cloak of Friendship." By Laurence Housman. (John Murray. 6s.)

### Notes and Queries.

**REGULATIONS.**

**The Editor is always willing to answer in this column, without any fee, all reasonable questions, as soon as possible.**

**But the following rules must be carefully observed.**

**1. Every communication must be accompanied by the name and address of the writer.**

**2. The question must always bear upon nursing, directly or indirectly.**

**If an answer is required by letter a fee of half-a-crown must be enclosed with the note containing the inquiry.**

*A Trained Nurse.*

(58) I was trained at Crumpsall Infirmary eighteen years ago, and am certificated for medical, surgical, and monthly nursing, and it was then a recognised training school. I am told now that it is not so, and that I am not entitled to call myself "a trained nurse."—*Nurse F.*

The training at Crumpsall Infirmary is excellent, and it is a recognised training school. There is no doubt that you are entitled to call yourself "a hospital-trained nurse."

*Workhouse Training.*

(59) Are there any nursing homes on the Continent at which nurses are taken who have had their training in workhouse hospitals (recognised training schools).—*J. T.*

The training you mention qualifies you for reception into any nursing home, here or abroad. Consult Burdett's "Hospitals and Charities," and write to the various homes asking as to vacancies.

*Surgical Work.*

(60) Can you inform me what is the shortest period and which is the hospital or infirmary I could get into for surgical work, and what terms? I am a certificated maternity nurse. *E. P.*

If you wish to go for a short time only, you must go as a paying probationer. Consult "How to become a Nurse," published by the Scientific Press, for full details.

*Massage.*

(61) Can you kindly give me the addresses of anyone near Bath giving lessons in massage from whom I could learn? Also, do you know of any hospital in London where a nurse who has just finished a provincial training could get a post as staff nurse or sister of out-patients' department?—*X. Y. Z.*

Write to the Hon. Secretary of the Bath Trained Nurses' Institute Home and Home for District Nurses, 44 and 45 Rivers Street, Bath. For your second question we can only advise you to advertise and answer advertisements.

*L.O.S.*

(62) How in London is it possible for a certified L.O.S. to get regular work or monthly nursing?—*A. B. T.*

By applying to medical men to whom you have an introduction or by the recommendation of patients, and also by advertising.

*Queen Alexandra's Imperial Military Nursing Service.*

(63) Can you tell me what course to take? I wish to qualify as a trained nurse with a view to ultimately becoming a member of Queen Alexandra's Imperial Military Nursing Service. I could not afford to pay a premium, but I could give twelve months' service. I am 22, and a farmer's daughter. I have tried a number of general hospitals without success. Should I be able to do as I wish if I got my training at a large union infirmary?—*M. P.*

Few general hospitals will take you as a probationer till you are 23 years of age. But you would find it exceedingly difficult to get on to the Military Nursing Service, as the nurses are almost limited to the daughters of professional men.

*Incurable.*

(64) Can you tell me the name of any home where a girl suffering from tuberculous trouble in the bones should be taken? The case is incurable and the girl has no money, but some might be collected if necessary. I do not think a home in London would be much use.—*L. R.*

You might find her eligible for the Brabazon Home of Comfort, Reigate. Write for particulars to the Hon. Secretary

**Handbooks for Nurses.**

| | Post Free. |
|---|---|
| "A Handbook for Nurses." (Dr. J. K. Watson.) ... | 5s. 4d. |
| "Nurses' Pronouncing Dictionary of Medical Terms" | 2s. 0d. |
| "Art of Massage." (Creighton Hale.) ... ... ... | 6s. 0d. |
| "Surgical Bandaging and Dressings." (Johnson Smith) | 2s. 0d. |
| "Hints on Tropical Fevers." (Sister Pollard.) ... | 1s. 8d. |

Of all booksellers or of The Scientific Press, Limited, 28 & 29 Southampton Street, Strand, London, W.C.

### For Reading to the Sick.

STRENGTH FOR TO-DAY.

"I think not of to-morrow,
Its trial or its task,
But still with childlike spirit
For present mercies ask.
With each returning morning
I cast old things away.
Life's journey lies before me:
My prayer is for to-day"

*Anon.*

It is wonderful how the Bible gives emphasis to this way of viewing life. When for forty years God fed his chosen people with bread from heaven, he never gave them, except on the morning before the Sabbath, more than one day's portion at a time. He positively forbade them gathering more than would suffice for the day, and if they should violate his command, what they gathered over the daily portion would become corrupt. Thus early God began to teach His people to live only by the day, and trust Him for to-morrow. At the close of the forty years, the promise given to one of the tribes was, "As thy days, so shall thy strength be."

Our Lord, also, in the form of prayer which He gave His disciples, taught this lesson of living by the day. There He has told us to ask for bread for one day only. "Give us this day our daily bread." Here, again, He teaches us that we have to do only with the present day. We do not need to-morrow's bread now: when we need it, it will be soon enough to ask God for it, and get it. It is the manna lesson over again. God is caring for us, and we are to trust Him for the supply of all our wants as they press upon us: we are to trust Him, content to have only enough in hand for the day.

God gives guidance by the day. One who carries a lantern at night does not see the whole path home; the lantern lights only a single step in advance; but, when that step is taken, another is thereby lighted, and so on until the end of the journey is reached. It is thus that God lights our way. He does not show us the whole of it when we set out: He makes one step plain, and then, when we take that, another and then another.

Every day brings its benedictions. If it is raining, rain is a blessing. If trouble comes, God draws nearer than before, for "as your days, so shall your strength be." Then in the trouble benedictions are folded up. If there is sorrow, comfort is revealed in the sorrow, a bright light in the cloud. If the day brings difficulties, hardships, heavy burdens, sharp struggles, life's best things come in just this kind of experience, and not in the easy ways. The thanksgiving heart finds treasure and good everywhere.

*Rev. J. R. Miller, D.D.*

"Why shouldst thou fill to-day with sorrow
About to-morrow,
My heart?
One watches all with care most true:
Doubt not that He will give thee, too,
Thy part."

*Anon.*

## Figures and Tables

**Figure f1:**